# MicroRNA-122 negatively associates with peroxiredoxin-II expression in human gefitinib-resistant lung cancer stem cells

**DOI:** 10.1038/s41417-018-0050-1

**Published:** 2018-10-19

**Authors:** Nisansala Chandimali, Do Luong Huynh, Jiao Jiao Zhang, Jae Cheol Lee, Dae-Yeul Yu, Dong Kee Jeong, Taeho Kwon

**Affiliations:** 1 Laboratory of Animal Genetic Engineering and Stem Cell Biology, Advanced Convergence Technology and Science, Jeju National University, Jeju, 63243 Republic of Korea; 2 Asan Institute for Life Sciences, Asan Medical Center, College of Medicine, University of Ulsan, Seoul, 05505 Republic of Korea; 3 Disease Model Research Laboratory, Genome Editing Research Center, Korea Research Institute of Bioscience and Biotechnology (KRIBB), Daejeon, 34141 Republic of Korea; 4 Laboratory of Animal Genetic Engineering and Stem Cell Biology, Subtropical/Tropical Organism Gene Bank, Jeju National University, Jeju, 63243 Republic of Korea

**Keywords:** Cancer stem cells, Non-small-cell lung cancer

## Abstract

Previously, we demonstrated that Prx II is important for survival of the gefitinib-resistant A549 (A549/GR) cell line, an NSCLC cell line derived by repeated exposure to gefitinib. Therefore, in this study, we used A549/GR cells to investigate the role of Prx II in GR NSCLC stemness. Initially, to explore the stemness characteristics and investigate the association of Prx II with those stemness characteristics, we successfully isolated a stem cell-like population from A549/GR cells. A549/GR CD133^+^ cells possessed important cancer stemness characteristics, including the abilities to undergo metastasis, angiogenesis, self-renewal, and to express stemness genes and epithelial–mesenchymal transition (EMT) markers. However, those characteristics were abolished by knocking down Prx II expression. MicroRNA 122 (miR-122) targets Prx II in A549/GR cancer stem cells (CSCs), thereby inhibiting the stemness characteristics in vitro and in vivo. Next, we investigate whether miR-122 overexpression was associated with Prx II expression and Prx-II-induced stemness characteristics, we transfected miR-122 into A549/GR CSCs. MiR-122 inhibited A549/GR stemness by downregulating the Hedgehog, Notch, and Wnt/β-catenin pathways. Taken together, our data suggest that Prx II promotes A549/GR stemness, and that targeting Prx II and miR-122 is a potentially viable strategy for anti-cancer-stem cell therapy in GR NSCLCs.

## Introduction

Peroxiredoxins (Prxs) comprise an important superfamily of cysteine (Cys)-based antioxidant enzymes, which are divided into three subclasses based on the number of conserved Cys residues participating in the redox reaction, i.e., the typical 2-Cys Prxs (Prxs I–IV), an atypical 2-Cys Prx (Prx V), and an atypical 1-Cys Prx (Prx VI) [1, 2]. These members of the Prx family have been frequently reported to be upregulated in many cancers, including breast, cervical, prostate, colorectal, mesothelioma, brain, and lung cancer [3–8]. Among the Prxs, Prx I, II, IV, and VI are aberrantly expressed with various potential effects on tumor progression in lung carcinomas, which is the leading cause of cancer-related death worldwide [9]. Previously, we showed the role of Prx II in a gefitinib-resistant (GR) A549 (A549/GR) non-small cell lung cancer (NSCLC) cell line, which was derived from the parental A549 cell line by repeated exposure to gefitinib [7]. NSCLC is one of the two main histological subtypes of lung cancers and represents most cases of lung cancer [10]. Aberrant expression of Prx II in NSCLCs has also been associated with induced tumor cell growth and proliferation via pJNK activation [7]. Furthermore, accumulating evidence has suggested that Prx II maintains cancer stem-like properties and induces the growth of colorectal cancer by activating the Hedgehog (HH) and Wnt/β-Catenin signaling pathways [11–13]. Prx II also maintains the stemness of hepatocellular carcinoma (HCC) stem cells via redox regulation [14]. Cancer stem cells (CSCs) are considered to be responsible for cancer progression, metastasis, and resistance to therapy [15]. Thus, in this study, we mainly focused on Prx II expression and Prx II-mediated stemness characteristics in A549/GR stem cells.

MicroRNAs (miRNAs) are small non-coding RNAs with the ability to regulate the expression of oncogenes, tumor suppressors, and numerous other genes and thereby influence the development of cancers [16]. Many recent studies have been aimed at developing identification systems for cancer-related miRNAs and their target genes, in order to elucidate the role of miRNAs in cancers [17]. Among them, miR-122 has been implicated as a tumor-suppressor gene in various types of cancers [18]. Recent studies have showed that miR-122 targets oncogenes, such as cyclin G1 and Bcl-2, thereby suppressing tumor proliferation [18, 19]. Overexpression of miR-122 in NSCLC cells induces chemo-sensitization for gemcitabine and radio-sensitization. Moreover, apoptosis and cell cycle arrest can be induced by miR-122 overexpression in NSCLC cells [19, 20]. Therefore, previous studies showed the potential application of miR-122 in NSCLC treatment. More importantly, one study demonstrated that miR-122 targets Prx II in HCC. MiR-122 downregulates Prx II expression by binding to Prx II and inhibits HCC cell growth by inducing apoptosis [21]. Here, we investigated the Prx II expression and mechanistic links that could explain the potential of Prx II in driving CSC properties, such as stemness, cell proliferation, metastasis, and angiogenesis in A549/GR stem cells. We also showed the direct effect of miR-122 in inhibiting Prx II expression. Thus, our findings provide new insights into the miR-122-mediated downregulation of A549/GR stem cell properties via Prx II inhibition.

## Materials and methods

### Cell culture, transfections, and generating stable cell lines

A549, A549/GR, A549/GR CD133^–^, A549 pCMV-Prx II, H460, H460/GR, HCC827, HCC827/GR cells, HCC827/GR shCON, and HCC827/GR shPrx II cells were grown in RPMI 1640 (Invitrogen, Carlsbad, CA, USA) supplemented with 10% fetal bovine serum (FBS: Hyclone, Logan, UT, USA), penicillin (100 U/ml) and streptomycin (100 mg/ml). A549/GR CD133^+^, A549/GR CD133^+^ shPrx II, and A549/GR CD133^+^ shCON cells were cultured in the above-mentioned complete medium supplemented with 10 ng/ml human epidermal growth factor (Sigma-Aldrich, St. Louis, LO, USA) and 20 ng/ml basic fibroblast growth factor (Koma Biotech, Daejeon, Korea). Transfection of the pCMV-Prx II vector and establishing the shPrx II and shCON cell lines were performed as described previously [7, 22]. MiR-122 was expressed from a DNA plasmid that was transfected using the Lipofectamine 2000 reagent (Invitrogen) according to manufacturer’s instructions. To screen for stable cell lines, the cells were plated in selective medium containing G418 (1000–2000 μg/ml, Invitrogen) for ~3 weeks, beginning at 48 h post transfection. The selective medium was replaced every 3 days. GFP-positive cells were selected.

### Magnetic-activated cell sorting to separate the CD133^+^ CSC population

A549/GR cells were separated on a magnetic-activated cell sorting column after labeling them with a primary anti-CD133 antibody (Miltenyi Biotech, Germany) according to the manufacturer’s instructions. The purity of the sorted CSCs was evaluated by western blotting.

### Cell proliferation and apoptosis assays

Cells were seeded at a density of 5 × 10^3^ cells/well in a 96-well plate, and the role of Prx II and miR-122 in A549/GR CSC proliferation was studied using the EZ-Cytox Kit (DoGenBio, Korea. Catalog number: EZ-3000), according to the manufacturer’s instructions. The absorbance in each well was measured at 450 nm. To detect apoptosis, cells were prepared using the Propidium Iodide (PI), Annexin V Detection Kit (BD Biosciences), per the manufacturer’s instructions, and analyzed by flow cytometry (FACSCalibur, BD Biosciences).

### Western blot analysis

Collected A549 and A549/GR cells were lysed in RIPA buffer (150 mM NaCl, 1% Nonidet p-40, 50 mM Tris, pH 8.0, and a protease inhibitor cocktail). Thirty micrograms of each sample was separated by SDS-PAGE and transferred to a nitrocellulose membrane (Bio-Rad, USA). Western blotting was performed with rabbit or mouse antibodies against Prx II: LF-PA0091 (AbFrontier, Seoul, South Korea), CD133: PA2049 (Boster Bio, CA, USA), Nanog: sc-293121, OCT3/4: sc-9081, Sox2: sc-365823 (Santa Cruz Biotechnology), VEGFR2: sc-6251, E-cad: sc-7870, Vimentin: sc-6260 (Santa Cruz Biotechnology), Shh: 2207 s, Gli-1: 3538 s, Notch 1: 3268 s (Cell Signaling Technology), CXCR4: YF-MA16239, STAT3: LF-MA30485,pSTAT3 (Tyr 705): LF-PA20474, pSTAT3 (Tyr-727): LF-PA20473 Hes-1: YF-MA11051, and β-Catenin: YF-MA10213 (AbFrontier). Protein expression levels were detected using Super signal West Pico PLUS Chemiluminescent Substrate (Thermo Fisher, #34577).

### DCF-DA assay

ROS levels in A549/GR shCON and shPrx II cells and A549/GR cells transfected with miR-122 and miR-NC were determined using DCF-DA (Invitrogen). Cells were incubated with 20 mM DCF-DA for 15 min at 37 °C and then washed with 1X PBS. Fluorescence due to DCF-DA deacetylation and oxidation was analyzed on a FACSCalibur flow cytometer (BD Biosciences).

### Transwell assays

Migration and invasion assays were performed using transwell 24-well chambers with 8.0-µM pore polycarbonate membranes (Merck Millipore, Darmstadt, Germany) without (migration) or with (invasion) Matrigel. Briefly, 200-µl volumes of A549/GR cell suspensions in medium containing 0.5% FBS were added separately to the upper chambers (1 × 10^5^ cells/chamber). In each case, the bottom chamber was filled with 800 µI of medium supplemented with 20% FBS as a chemoattractant. The cells were then incubated for 24 h at 37 °C in 5% CO_2_. Cells that passed through the coated membrane to the lower surface were then fixed with 4% paraformaldehyde and stained with 0.1% crystal violet for 1 h. Images were captured using a microscope.

### Wound-healing assay

A549/GR CD133^+^ and CD133^−^ cells were plated in a six-well plate at 1 × 10^6^ cells/well and allowed to grow into a monolayer. At 90% confluence, the complete medium was replaced with serum-free medium and the cells were cultured overnight until a complete monolayer formed. Using a wound maker (Essen Bioscience, Ann Arbor, MI 48108, USA), a linear scratch was made in the cell monolayer. Cells were rinsed with 1X PBS and fresh complete media was added. Photomicrographs were taken of the migrated cells at 0 and 24 h using the IncuCyte Live-Cell Analysis System (IncuCyte).

### Sphere-formation and ICC assays

A549/GR shCON, shPrx II, CD133^+^, and CD133^−^ cells (2 × 10^3^ cells/well) were seeded in a six-well Ultra Low Cluster plate (Corning Inc., Corning, NY, USA) and cultured for 10 days in suspension in serum-free Dulbecco’s modified Eagle’s medium/F12 (Gibco, Grand Island, NY, USA) containing B27 supplement (1: 50; Invitrogen), 20 ng/ml EGF (Calbiochem, CA, USA), and 0.5% bovine serum albumin (Sigma-Aldrich). The spheres were imaged with an inverted microscope and counted. Sphere-formation efficiency was calculated as the number of colonies/input cells × 100%. ICC staining of spheres was performed following the steps of the ICC assay, as described below.

### ICC assay

A549 cells and A549/GR cells were fixed with 3.7% formaldehyde in 1X PBS for 10 min at room temperature. They were then blocked with 1X PBS containing 0.5% Triton X-100 and 1% bovine serum for 60 min at room temperature and incubated with primary antibodies for 18 h. On the following day, the cells were washed with 1X PBS with Tween 20 and incubated with secondary antibodies for 2 h at room temperature. Nuclei were visualized by 4’,6-diamidino-2-phenylindole (DAPI) staining. Nuclear staining was observed qualitatively under a microscope after 20 min of DAPI staining, and images were acquired.

### Colony-formation assay

A549/GR CD133^–^, A549/GR CD133^+^ shCON, shPrx II, miR-122, and miR-NC-transfected cells (1 × 10^3^ cells/well) were plated in six-well plates and maintained in an incubator at 37 °C with 5% CO_2_ for 7 days. Cells were then washed with 1X PBS, fixed with 3.7% formaldehyde for 10 min, treated with methanol for 20 min, and stained with crystal violet for 30 min. The plates were washed three times with 1X PBS prior to image capturing.

### Xenograft model and optical imaging

Mice were maintained and used for experiments, according to a protocol approved by the Institutional Animal Care and Use Committee of Jeju National University (Jeju, South Korea). The tumorigenicity of A549/GR CD133^+^ cells transfected with miR-122 or miR-NC was assayed after subcutaneously inoculating 1 × 10^5^ cells in a mixture of 100 µl Matrigel (Sigma-Aldrich) and 1X PBS into the flanks of 8-week-old athymic BALB/c female nude mice (*n* = 5/group). Tumor growth was observed at 35 days post inoculation by IRDye® 800CW 2-DG (radiolabeled 2-deoxy-D-glucose [2-DG])-based optical imaging of xenograft models.

### Immunofluorescence

Paraffin-embedded tumor tissues were cut in 4-μm sections, deparaffinized in xylene, and rehydrated through a graded ethanol series. Next, the sections were rinsed three times in 1X PBST (PBS with Tween 20). The sections were then blocked in 1X PBS containing 0.5% Triton X-100 and 5% sheep serum for 30 min at room temperature before incubating them overnight at 4 °C with a rabbit anti-Prx II polyclonal antibody (AbFrontier technology, Seoul, South Korea) and a mouse anti-vimentin monoclonal antibody (Santa Cruz Biotechnology). Sections were again rinsed three times in 1X PBST. To detect the bound primary antibodies, the sections were incubated in the dark at room temperature for 90 min with goat anti-rabbit and goat anti-mouse antibodies (Santa Cruz Biotechnology). Slides were counterstained for 20 min in the dark with DAPI diluted in 1X PBS before visualization and image capturing under a microscope.

### Data analysis

All experiments were repeated five times, and data are presented as the mean ± standard error. Statistical analyses were performed using the Statistical Package for the Social Sciences (SPSS version 20.0.1; SPSS, Chicago, IL, USA). Statistically significant difference among treatment groups were determined via one-way ANOVA and Fisher’s least significant difference (LSD) test. *P*-values <0.05 are considered significant.

## Results

### Prx II expression mediated the enhanced stemness of GR NSCLC cells

**Fig. 1:**
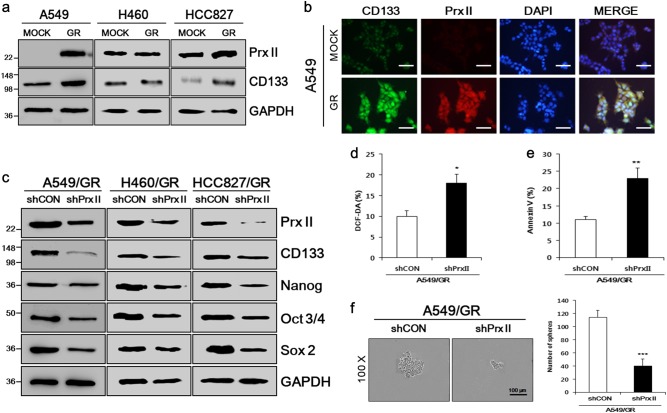
Prx II expression and its significance in GR NSCLC. **a** Expression of Prx II and the stem cell marker CD133 was detected in A549, H460, and HCC827 cells and the derived GR cells (A549/GR, H460/GR, HCC827/GR) by western blotting. **b** Prx II and CD133 expression were confirmed in A549 and A549/GR cells by performing ICC assays. **c** The effects of knocking down Prx II on the expression of CD133 and the stemness-related genes Nanog, Oct3/4, and Sox2 in A549/GR, H460/GR, and HCC827/GR cells were detected by western blotting. **d** DCF-DA assays were performed to detect the ROS levels in A549/GR shCON and A549/GR shPrx II cells. **e** Apoptotic cell populations were detected by Annexin V staining. **f** Sphere-formation assays were performed to observe the effect of Prx II knockdown on sphere-formation in A549/GR cells. Differences in the sphere size and number of spheres were detected in A549/GR shCON and shPrx II cells. Data represent mean ± SEM (*n* = 5 per group). *P < 0.05, **P < 0.01, ***P < 0.001. Bar represents 100 microns

Prx II is highly expressed in A549/GR cells, but not in parental A549/MOCK cells [7]. Western blot analysis was performed to verify that Prx II was expressed in A549/GR cells and to observe Prx II expression in other GR NSCLC cells. As expected, Prx II was expressed in A549/GR cells, but not in A549/MOCK cells. Other NSCLC cell lines (MOCK) and their GR variants showed relatively similar Prx II-expression levels. Parallel experiments showed that higher expression of the CSC marker, CD133 was present in GR NSCLC cells, compared with MOCK cells (Fig. 1a). High expression of Prx II and CD133 in A549/GR cells was confirmed by performing immunocytochemistry (ICC) assays (Fig. 1b).

To examine the relationship between Prx II expression and the enhanced stemness of GR NSCLC cells in greater detail, we generated cell lines expressing short hairpin RNAs (shRNAs) against Prx II or a control target. GR-shPrx II (Prx II knockdown) and GR-shCON (control) variants were established with the A549, H460, and HCC827 cell lines, as described in our previous report [7]. Western blot assays validated the successful knockdown of Prx II in GR-shPrx II cells and showed that stemness was inhibited in GR NSCLC cells by Prx II knockdown, as observed by the reduced expression of CD133 and the stemness-related genes Nanog, Oct3/4, and Sox2 in GR-shPrx II, compared with GR-shCON cells (Fig. 1c).

Because, we observed significantly different Prx II expression in A549/MOCK and A549/GR cells compared with other NSCLC cell lines, we selected A549/GR cells for further experiments. We studied the effect of Prx II knockdown on reactive oxygen species (ROS) levels by performing dichloro-dihydro-fluorescein diacetate (DCF-DA) assays and measuring apoptosis by Annexin V staining. Increased percentages of DCF-DA- and Annexin V-positive A549/GR-shPrx II cells showed the induction of ROS levels and apoptosis by Prx II knockdown (Fig. 1d, e). Prx II knockdown suppressed the self-renewal capacity of A549/GR cells, as observed by diminished sphere-formation capacity in A549/GR-shPrx II, compared with A549/GR-shCON cells (Fig. 1f). These observations imply that Prx II mediates the stemness of A549/GR cells.

### Cancer stem-like properties were exhibited in A549/GR CD133^+^ cells with high Prx II expression

**Fig. 2:**
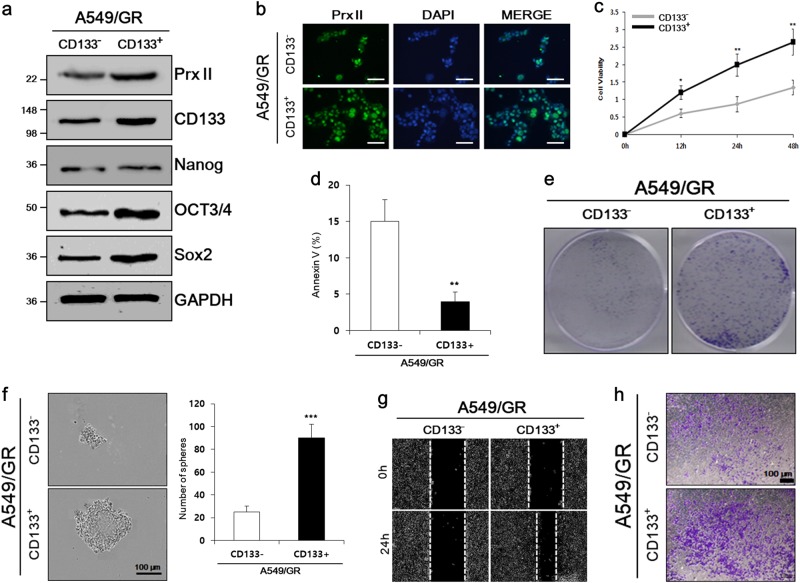
A549/GR CD133^+^ cells possessed stemness characteristics. **a** Western blot analysis was performed to check the expression levels of Prx II, CD133, Nanog, Oct3/4, and Sox2 in A549/GR CD133^−^ and A549/GR CD133^+^ cells. **b** Higher expression levels of Prx II in A549/GR CD133^+^ cells than in CD133^–^ cells was verified by ICC. **c** Proliferation of A549/GR CD133^+^ and CD133^–^ cells was measured by performing MTT assays. **d** Annexin V staining was conducted to measure apoptosis in A549/GR CD133^–^ and CD133^+^ cells. **e** The ability of A549/GR CD133^+^ and CD133^−^ cells to form colonies (i.e., the self-renewal ability) was compared by performing colony-formation assays. **f** The sphere-forming ability was detected in A549/GR CD133^+^ and CD133^–^ cells by observing the sizes and numbers of spheres in sphere-forming assays. **g** Wound-healing assays were performed to check the cell-migration abilities of A549/GR CD133^+^ and CD133^–^ cells. **h** Transwell invasion assays were performed to compare the invasive abilities of A549/GR CD133^+^ and CD133^−^ cells. Data represent mean ± SEM (*n* = 5 per group). *P < 0.05, **P < 0.01, ***P < 0.001. Bar represents 100 microns

The cell surface marker, CD133 has been used to isolate CSC (CD133^+^ cell) populations [23]. Our above results showed that some A549/GR cells expressed CD133. We separated the CD133^+^ subpopulation of A549/GR cells (A549/GR CD133^+^) from the A549/GR CD133^–^ cells. Both subpopulations were examined for their cancer stem-like properties. High Prx II expression was observed in A549/GR CD133^+^ cells compared with A549/GR CD133^–^ cells by western blotting (Fig. 2a) and ICC assays (Fig. 2a, b). Furthermore, western blotting showed the enhanced expression of CD133 and stemness-related genes Nanog, Oct3/4, and Sox2 in CD133^+^ cells compared with CD133^–^ cells, in parallel with high Prx II expression (Fig. 2a). These data showed that the enhanced stemness of CD133^+^ cells correlated with high Prx II expression.

CSCs are characterized by high potentials for self-renewal, differentiation, and metastasis [24]. Therefore, we further examined A549/GR CD133^+^ cells for those stem-like properties. We performed 3-(4,5-dimethylthiazol-2-yl)-2,5-diphenyltetrazolium bromide (MTT) assays and Annexin V staining to assess the proliferation of CD133^+^ cells. Increased viability and reduced numbers of Annexin V-positive CD133^+^ cells (versus CD133^–^ cells) indicated that proliferation was enhanced due to inhibited apoptosis (Fig. 2c, d). CD133^+^ cells showed greater colony-formation ability in vitro than CD133^–^ cells, confirming the enhanced cell proliferation of CD133^+^ cells (Fig. 2e). Increased self-renewal and differentiation abilities were observed through the larger size and increased number of CD133^+^ spheres in sphere-formation assays (Fig. 2f).

Next, wound-healing assays revealed that CD133^+^ cells were capable of significantly faster wound closure than CD133^–^ cells (Fig. 2g), and a transwell assay with Matrigel demonstrated that CD133^+^ cells were more invasive than CD133^–^ cells (Fig. 2h), indicating the higher metastatic capacity of CD133^+^ cells (as cell-migration ability is required for cancer metastasis) [25]. Taken together, these results confirmed A549/GR CD133^+^ cells had stem cell characteristics and high-level Prx II expression.

### Prx II knockdown diminished cell proliferation, metastasis, and angiogenesis in A549/GR CSCs

**Fig. 3:**
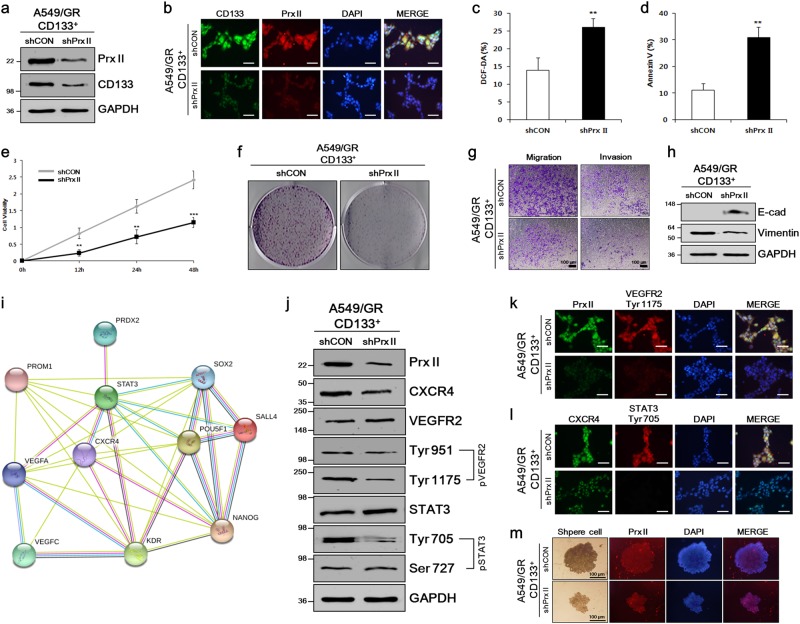
Prx II was associated with stemness characteristics in A549/GR CSCs. **a** Prx II knockdown in A549/GR CD133^+^ cells was validated by western blot and **b** ICC analysis. Reduced CD133 expression in A549/GR CD133^+^ shPrx II cells compared with A549/GR CD133^+^ shCON cells was also detected. **c** DCF-DA assays were conducted to compare ROS levels in shCON and shPrx II cells. **d** The percentages of apoptotic cells in shCON and shPrx II cells were determined by Annexin V staining. **e** An MTT assay comparing the proliferation of shCON and shPrx II cells. **f** Colony-formation assay showing reduced colony formation of shPrx II cells versus shCON cells. **g** Transwell migration and invasion assays were used to compare the abilities of shCON and shPrx II cells to migrate and invade. **h** Western blotting against E-cadherin and vimentin expression was used to evaluate metastasis in shPrx II and shCON cells. **i** Prx II interaction with genes related to stemness and angiogenesis was visualized using STRING software. **j** The production levels of angiogenesis-related genes (CXCR4, VEGFR2, pVEGFR2, STAT3, and pSTAT3) were examined in shCON and shPrx II cells by western blot analysis and **k**, **l** ICC. **m** A sphere ICC assay was performed to study the sphere-formation ability and CXCR4 expression in shCON and shPrx II spheres. Data represent mean ± SEM (*n* = 5 per group). **P < 0.01, ***P < 0.001. Bar represents 100 microns

We then investigated whether Prx II is involved with the stem-like properties of A549/GR CSCs. Thus, we established Prx II-knockdown A549/GR CD133^+^ (shPrx II) and control A549/GR CD133^+^ (shCON) cell lines, as described in our previous study [7], and studied the effects of Prx II knockdown on A549/GR CSCs. Successful knockdown of Prx II was initially validated by western blot analysis and ICC assays. CD133 expression also was reduced in shPrx II cells compared with shCON cells, indicating the reduction of the CD133^+^ CSC population by Prx II knockdown (Fig. 3a, b). ROS levels and the proportion of Annexin V-positive cells increased in shPrx II cells compared with shCON cells, indicating that apoptosis was induced by Prx II knockdown (Fig. 3c, d). Reduced shPrx II cell proliferation (compared with that of shCON cells) was observed in MTT and colony-formation assays (Fig. 3e, f). These data indicated that Prx II knockdown negatively associated with A549/GR CSC proliferation.

To gain insight into how Prx II knockdown alters cancer cell metastasis, we performed transwell assays (Fig. 3g). Both the migration and invasion capacities were reduced by Prx II knockdown, compared with those of shCON cells. Furthermore, higher expression of the epithelial marker E-cadherin and lower expression of the mesenchymal marker Vimentin in shPrx II cells (relative to that in shCON cells) indicated the suppressing Prx expression inhibited the epithelial–mesenchymal transition (EMT) (Fig. 3h). Collectively, these results demonstrated that in vitro Prx II knockdown abolished the enhanced metastatic capacity of A549/GR CSCs.

To study the effects of Prx II knockdown on A549/GR CSC angiogenesis, we predicted the Prx II-interaction network with respect to stemness-related and angiogenesis-related genes using STRING software (Fig. 3i). Based on the STRING results, we checked the levels of C-X-C chemokine receptor type 4 (CXCR4), vascular endothelial growth factor receptor 2 (VEGFR2), phosphorylated VEGFR2 (pVEGFR2), signal transducer and activator of transcription 3 (STAT3), and phosphorylated STAT3 (pSTAT3) in shPrx II, compared with shCON cells. Our results showed the reduction of CXCR4 expression in shPrx II cells. In addition, the phosphorylation of VEGFR2 (Tyr 951, Tyr 1175) and STAT3 (Tyr 705) were inhibited in shPrx II cells, compared with that in shCON cells. However, phosphorylation at the Ser 727 site was not inhibited by Prx knockdown (Fig. 3j). ICC assays further confirmed the reduction of Prx II, VEGFR2 (Tyr 1175), CXCR4, and STAT3 (Tyr 705) production in shPrx II cells, compared with shCON cells(Fig. 3k, l). Moreover, the sphere ICC assay revealed a reduced sphere-formation ability and Prx II expression after Prx II knockdown (Fig. 3m). Together, these data indicated that Prx II negatively regulated A549/GR CSC proliferation, metastasis, and angiogenesis.

### miR-122 mimic transfection suppressed cellular proliferation, metastasis, and angiogenesis of A549/GR CSCs by inhibiting Prx II

**Fig. 4:**
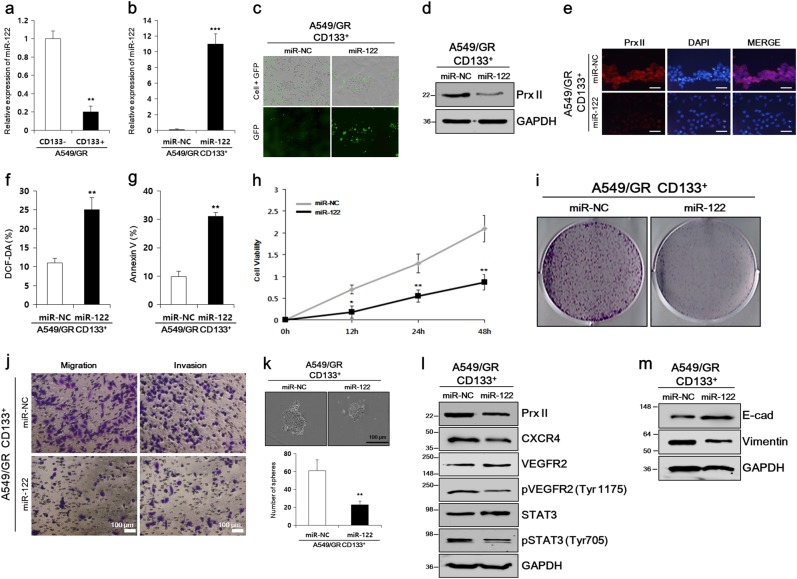
MiR-122 targeted Prx II and Prx II-induced stemness characteristics in A549/GR CSCs. **a** Relative miR-122 expression in A549/GR CD133^+^ and A549/GR CD133^–^ cells was checked by real-time PCR analysis. **b** Transfection of miR-122 into A549/GR CD133^+^ cells was validated by real-time PCR analysis. **c** GFP-positive cells were screened in miR-122- and miR-NC-transfected A549/GR CD133^+^ cells. **d**, **e** Western blot and ICC assays were performed to check the Prx II-expression levels in miR-NC- and miR-122-transfected cells. **f** DCF-DA assay results showing the induced ROS level in A549/GR cells after miR-122 transfection. **g** An increased percentage of apoptotic cells was observed in miR-122-transfected A549/GR CD133^+^ cells by Annexin V staining. **h** Cell proliferation was observed by performing MTT assays with miR-122- and miR-NC-transfected cells. **i** Inhibited colony-formation ability of miR-122-transfected A549/GR CD133^+^ cells, as determined by performing colony-formation assays. **j** The migration and invasion abilities of miR-122- and miR-NC-transfected cells were determined in Transwell migration and invasion assays. **k** Reduced sphere-formation ability of miR-122-transfected A549/GR CD133^+^ cells compared with miR-NC-transfected cells. **l** Expression of angiogenesis-related genes. **m** EMT-related expression markers were compared in miR-122- and miR-NC-transfected cells by western blot analysis. Data represent mean ± SEM (*n* = 5 per group). *P < 0.05, **P < 0.01. Bar represents 100 microns

Prx II is a direct target of miR-122 in HCC [21]. Therefore, we studied whether miR-122 targets Prx II in A549/GR CSCs. We thus measured the baseline expression of endogenous miR-122 in A549/GR CD133^+^ and CD133^–^ cells. Real-time PCR showed that miR-122 expression was lower in CD133^+^ cells than in CD133^−^ cells (Fig. 4a). We then prepared separate A549/GR CD133^+^ cell lines stably expressing miR-122 or miR-NC. MiR-122 expression was quantified by real-time PCR 3 days after transfection. Increased miR-122 expression validated the successful transfection (Fig. 4b). The miR-122 transfection was also evaluated by quantifying the percent of green fluorescent protein (GFP)-positive cells (Fig. 4c). Then, we checked whether miR-122 overexpression inhibited Prx II expression in A549/GR CSCs. As expected, the transfection of miR-122 substantially downregulated the Prx II expression in A549/GR CSCs, as evidenced by western blot analysis and ICC assays (Fig. 4d, e).

Next, we compared miR-122- and miR-NC-transfected A549/GR CSCs to gain further insight into how miR-122 affects CSC properties by inhibiting Prx II. MiR-122 increased the percentages of DCF-DA and Annexin V-positive cells (Fig. 4f, g) and reduced the time-dependent viability of A549/GR CSCs (Fig. 4h), revealing that miR-122-induced cell death by inhibiting Prx II in A549/GR CSCs. A colony-formation assay showed that miR-122 reduced the colony-forming ability and, thus, the proliferation of A549/GR CSCs (Fig. 4i). Transwell assays with or without Matrigel showed that miR-122 abolished the high invasiveness and migration abilities of A549/GR CSCs (Fig. 4j). Furthermore, smaller spheres and fewer spheres observed in sphere-formation assays indicated the reduction of the self-renewal ability of A549/GR CSC after miR-122 transfection (Fig. 4k). Similar to the effects of Prx II knockdown, western blot analysis showed that miR-122 transfection downregulated CXCR4 expression, as well as VEGFR2 (Tyr 1175) and STAT3 (Tyr 705) phosphorylation, indicating that angiogenesis was inhibited (Fig. 4l). Western blotting also indicated that increased E-cadherin expression and decreased vimentin expression after miR-122 transfection inhibited the EMT in A549/GR CSCs (Fig. 4m). Taken together, these findings suggested that miR-122 suppressed the CSC properties of A549/GR cells by inhibiting Prx II.

### Transfection of miR-122 led to more profound inhibitory effects of tumor growth in vivo via Prx II inhibition

**Fig. 5:**
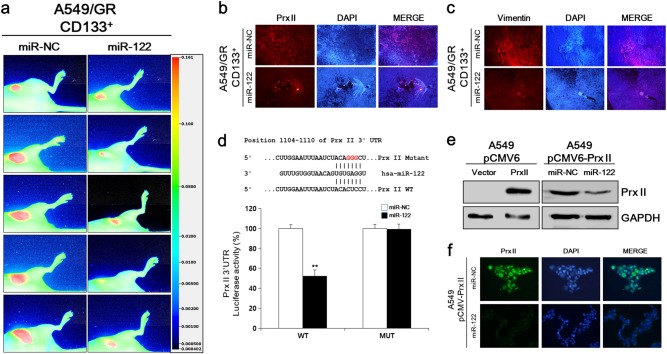
MiR-122 directly targeted Prx II and inhibited tumor growth in vivo. **a** Growth of tumors grown from miR-122- and miR-NC-transfected A549/GR CD133^+^ cells was determined by the IRDye® 800CW 2-DG (radiolabeled 2-deoxy-D-glucose [2-DG])-based optical imaging of xenograft models. **b**, **c** Immunofluorescence analysis of Prx II and vimentin expression in miR-122- and miR-NC-transfected A549/GR CD133^+^ tumor tissues. **d** Luciferase assays were conducted to characterize the predicted binding site of miR-122 in Prx II mRNA. **e** Prx II expression was studied in lysates of A549 cells and Prx II-overexpressing A549 cells by immunoblotting or (**f**) by conducting ICC assays with an anti-Prx II antibody. Data represent mean ± SEM (*n* = 5 per group). **P < 0.01. Bar represents 100 microns

In vivo studies were conducted to evaluate the effects of miR-122 on A549/GR CSCs in more depth. As shown in Fig. 5a, miR-122 significantly decreased tumor growth, as observed by the smaller tumor sizes of miR-122-transfected tumors compared to the miR-NC-transfected tumors. Immunofluorescence assays showed that Prx II and vimentin expression levels were reduced in vivo in miR-122-transfected tumor tissues compared with miR-NC-transfected tumor tissues, indicating that miR-122 targets Prx II and inhibits the EMT in A549/GR tumors (Fig. 5b, c).

To elucidate the molecular mechanisms by which miR-122 executes its function, we used a Prx II 3′-untranslated region (UTR) luciferase reporter assay. As shown in Fig. 5d, Prx II 3′-UTR luciferase reporter activity was reduced by miR-122, and that reduction was abolished by mutation of the Prx II 3′-TR. Next, we established an A549 pCMV6-Prx II cell line overexpressing Prx II to study the effects of miR-122 on Prx II overexpression in A549 cells. The A549 pCMV6-Prx II cell line was generated as described previously [22]. Then, miR-122 and miR-NC were separately transfected into Prx II-overexpressing cells, and Prx II expression was checked by western blotting. As shown in Fig. 5e, Prx II expression was increased in pCMV6-Prx II cells compared to cells transfected with the empty vector, indicating the successful overexpression of Prx II. However, Prx II expression was downregulated in miR-122-transfected cells compared with miR-NC-transfected cells, indicating that miR-122 inhibited the Prx II expression in A549 cells. An ICC assay further confirmed that miR-122 inhibited Prx II expression (Fig. 5f). These data indicated that miR-122 inhibited tumor growth by directly targeting Prx II in vivo.

### MiR-122 targeted HH, Notch, and Wnt/β-Catenin signaling in A549/GR CSCs by inhibiting Prx II

**Fig. 6:**
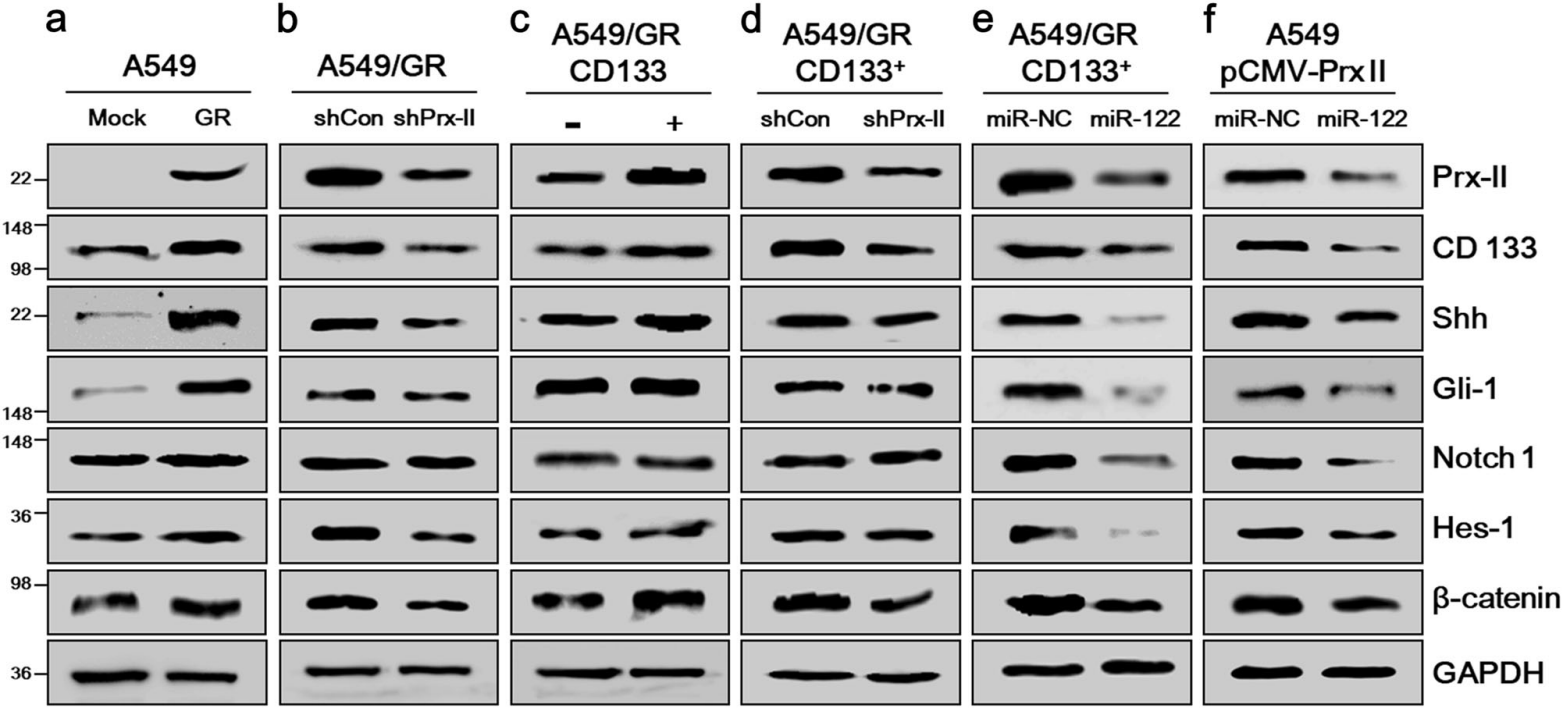
Profound inhibitory effects of miR-122 on the HH, Notch, and Wnt/β-Catenin pathways by downregulating Prx II. **a** Western blots were performed to detect the expression levels of Prx II, CD133, HH signaling-related markers, Shh, and Gli-1, Notch signaling-related markers (Notch 1 and Hes-1), and β-Catenin in A549/MOCK and A549/GR cells. **b** A549/GR shCON and shPrx II cells, **c** A549/GR CD133^+^ and CD133^−^ cells, **d** A549/GR CD133^+^ shCON and shPrx II cells, **e** A549/GR CD133^+^ cells transfected with miR-NC or miR-122, and **f** Prx II-overexpressing A549 (pCMV-Prx II) cells transfected with miR-NC and miR-122

We conducted a series of western blot assays to study the effects of Prx II expression on the HH, Notch, and Wnt/β-Catenin signaling pathways in A549/GR CSCs. As shown in Fig. 6a, the expression levels of CD133, HH signaling-related genes (Shh, Gli-1), Notch signaling-related genes (Notch-1, Hes-1), and β-Catenin were higher in A549/GR cells than in A549/MOCK cells. Those higher expression levels were abolished by Prx II knockdown in A549/GR cells (Fig. 6b). As shown in Fig. 6c, the expression levels of CD133, Shh, Hes-1, and β-Catenin were higher in CD133^+^ cells than in CD133^–^ cells. However, the expression levels of Gli-1 and Notch 1 did not show significant differences between CD133^+^ and CD133^–^ cells (Fig. 6c).

Next, we utilized A549/GR CD133^+^ shPrx II cells (compared to shCON cells) and miR-122-transfected A549/GR CD133^+^ cells (compared to miR-NC-transfected A549/GR CD133^+^) cells to study the effects of Prx II on the expression of CD133, HH, and Notch signaling-related genes and β-Catenin. CD133 expression was reduced in shPrx II cells compared with shCON cells and in miR-122-transfected cells compared with miR-NC-transfected cells. The Shh and Gli-1 expression levels were slightly reduced, and Notch 1 and Hes-1 were expressed similarly in shPrx II cells compared with shCON cells. Interestingly, the expression levels of Shh, Gli-1, Notch 1, and Hes-1 decreased significantly in miR-122-transfected A549/GR CD133^+^ cells compared with miR-NC-transfected cells and A549/GR CD133^+^ shPrx II cells. β-Catenin expression was downregulated in both shPrx II cells and miR-122-transfected cells, compared with shCON cells and miR-NC-transfected cells (Fig. 6d, e). These data suggested that the inhibition of Prx II expression by miR-122 transfection had more profound inhibitory effects on signaling pathways than did Prx II knockdown in A549/GR CSCs. Finally, we used A549 pCMV6-Prx II cells to study the effects of miR-122 on the aforementioned signaling pathways in A549 cell via Prx II inhibition. Expression of Prx II, CD133, Shh, Gli-1, Notch 1, Hes-1, and β-Catenin decreased in miR-122-transfected A549 pCMV6-Prx II cells (Fig. 6f). Together, these results indicated that miR-122 induced Prx-II-mediated inhibition of the HH, Notch, and Wnt/β-Catenin signaling pathways in A549 cells.

## Discussion

NSCLC is the most common histological form of lung cancer and accounts for 80% of all cases [26]. Previously, we reported the expression levels of Prx isotypes in different NSCLC cell lines resistant to epidermal growth factor receptor-specific tyrosine kinase inhibitors (EGFR-TKIs) [7]. All six Prx isotypes were stably expressed in NSCLC cell lines, except that in A549 cells. Because, Prx II was highly expressed in A549/GR cells, but not in parental A549 cells or other EGFR-TKI-resistant A549 cells due to the methylation of 5′-CCGG-3′ site, the upstream of Prx II gene [7]. Gefitinib interrupts signaling through the EGFR in target cells [27]. Initially, we verified the Prx II-expression levels in three different NSCLC cell lines (A549, H460, and HCC827), along with GR variants of these cell lines. We selected the A549/GR cell line for this study because of the significant difference of Prx II expression, compared with the parental A549 cells. We also showed how Prx II knockdown downregulated the stemness and cell proliferation of NSCLC cells.

As slowly dividing cells with unlimited proliferative potential, CSCs have been found to be responsible for NSCLC formation and a failure to respond to current treatments, including gefitinib [28, 29]. Therefore, we sought to determine whether and how Prx II expression associates with the CSC properties of A549/GR cells. First, we isolated A549/GR stem cells as a subpopulation of cells expressing the CD133 marker (A549/GR CD133^+^). CD133 is a cell-surface transmembrane glycoprotein that is widely used as a stem cell marker in a variety of normal and tumor tissues [30, 31]. In this study, we found that Prx II expression was higher in CD133^+^ A549/GR stem cells than in CD133^–^ A549/GR stem cells. Our data clearly showed that the A549/GR CD133^+^ cell possessed cancer stem-like properties, such as high stemness, cell proliferation, self-renewal ability, and metastasis, indicating that the CSCs were successfully isolated. Therefore, CD133^+^ cells with high Prx II expression were used for further experiments.

Prx II-knockdown A549/GR CD133^+^ (A549/GR CD133^+^ shPrx II) cells were used to ascertain the effect of Prx II knockdown on A549/GR CSCs. A549/GR shCON cells were used as the control after stably transfection with a scrambled shRNA. First, we validated the successful knockdown of Prx II in A549/GR stem cells. Moreover, our data showed that CD133 expression was also downregulated by Prx II knockdown in CSCs. Apoptosis and ROS levels were increased in shPrx II cells. However, the capacities of A549/GR CD133^+^ cells to proliferate, migrate, invade, and undergo the EMT were inhibited by Prx II knockdown, indicating that Prx II knockdown reduced the metastatic potential. EMT (the loss of the epithelial cell phenotype and the acquisition of the mesenchymal phenotype) is considered a cellular program that modifies tumor cells to facilitate metastasis [32]. Further supporting the role of Prx II in A549/GR stem cells, we demonstrated that Prx II-induced VEGF signaling, as Prx II knockdown inhibited VEGFR2 and STAT3 phosphorylation. In the absence of Prx II, VEGFR2 becomes inactive and no longer responds to VEGF stimulation [33]. Previous reports have shown that the inhibition of STAT3/VEGFR2/VEGF signaling is involved in the anti-angiogenic effects in NSCLC cells [34]. Our previous study also showed that Prx II was positively associated with STAT3/VEGFR2/VEGF signaling and, thus, with angiogenesis in HCC [8, 14]. Thus, our results indicated that Prx II knockdown inhibits angiogenesis in A549/GR CSCs, considering that VEGF and its signaling pathway play a dominant role in angiogenesis [35]. Angiogenesis is one of the most important phenomena involved in sustaining tumor development, proliferation, and metastasis [25]. Prx II was identified as a direct target of miR-122, which induces apoptosis and inhibits the proliferation of HCC cells by inhibiting Prx II [21]. We attempted to determine whether miR-122 associates with high Prx II expression in A549/GR stem cells. We observed a lower baseline level of miR-122 expression in CD133^+^ cells than in CD133^–^ cells. Therefore, we hypothesized that the lower level of miR-122 expression may be related to higher Prx II expression in A549/GR CSCs. To test this hypothesis, we transfected miR-122 and miR-NC separately into CD133^+^ cells. Prx II expression was reduced in the miR-122-transfected cells, compared with the miR-NC (control)-transfected cells, demonstrating that miR-122 overexpression inhibited Prx II in A549/GR stem cells. Moreover, our results showed that inhibiting Prx II expression by miR-122 abolished the stem cell properties driven by higher Prx II expression in A549/GR stem cells, including cell proliferation, migration, invasion, EMT, self-renewal ability, and angiogenesis. In support of our hypothesis, our in vivo results showed reduced Prx II expression and tumor growth in miR-122-transfected CD133^+^ cells. In this study, we found that miR-122 inhibits luciferase activity from the 3′-UTR of Prx II mRNAs. Prx II is not expressed in A549 cells [7]. Thus, to study the effect of miR-122 on Prx II expression in A549 cells, we established a Prx II-overexpressing A549/pCMV6-Prx II cell line. Our results confirmed that miR-122 inhibits Prx II expression in A549 cells. Aberrantly activated HH signaling has been found to drive CSC properties in various types of cancer [36]. However, the role of HH in NSCLC remains controversial, and some studies have reported three subtypes of NSCLCs, depending on the HH activity. A549 cells have high HH activity and are insensitive to existing HH inhibitors due to high Gli-1 expression [37]. Notch signaling promotes proliferation and inhibits apoptosis in NSCLC cells, thereby playing a critical role in tumorigenesis and drug resistance [38]. Upregulation of the Wnt/β-catenin signaling pathway has been reported to activate genes involved in NSCLC cell proliferation, metastasis, and angiogenesis [39]. Furthermore, previous data suggested that Prx II plays a crucial role in activating the HH and Wnt/β-catenin signaling pathways in colorectal cancer cells [11, 12]. Therefore, we studied the association of Prx II with the aforementioned signaling pathways in A549/GR cells and stem cells. Our results clearly showed that miR-122 targeted Prx II mRNA and inhibited the HH, Notch, and Wnt/β-catenin signaling pathways in A549/GR stem cells and A549 cells overexpressing Prx II. Interestingly, these signaling pathways were not significantly inhibited by Prx II knockdown in A549/GR stem cells. Our results indicated that miR-122 has profound inhibitory effects on the HH, Notch, and Wnt/β-catenin signaling pathways and, thus, the stemness characteristics of A549/GR stem cells, compared with Prx II-knockdown A549/GR cells.

In conclusion, we found that Prx II overexpression could be critical for the cancer stem-like properties of A549/GR CSCs. In addition, Prx II-induced stemness characteristics were abolished by miR-122 via Prx II targeting. Therefore, restoring miR-122 expression in A549/GR stem cells may an effective in the therapy against NSCLCs.
